# Developing broad-spectrum resistance in cassava against viruses causing the cassava mosaic and the cassava brown streak diseases

**DOI:** 10.3389/fpls.2023.1042701

**Published:** 2023-01-26

**Authors:** Samar Sheat, Stephan Winter

**Affiliations:** Plant Virus Department, Leibniz Institute DSMZ-German Collection of Microorganisms and Cell Cultures, Braunschweig, Germany

**Keywords:** resistant germplasm, precise virus screening, disease tolerance, plant immunity, resistant cassava, dual virus resistance

## Abstract

Growing cassava in Africa requires resistance against the viruses causing cassava mosaic disease (CMD) and the viruses causing cassava brown streak disease (CBSD). A dominant CMD2 resistance gene from a West African cassava landrace provides strong resistance against the cassava mosaic viruses. However, resistance against cassava brown streak viruses is limited to cassava varieties that show tolerance to the disease. A recently identified cassava germplasm that cannot be infected with cassava brown streak viruses provides a new source of the resistance required to protect cassava from CBSD. We present a synopsis of the status of virus resistance in cassava and report on the research to combine resistance against CBSD and CMD. We improve the lengthy and erratic screening for CBSD resistance by proposing a virus infection and screening protocol for the viruses causing CBSD and CMD, which allows a rapid and precise assessment of cassava resistance under controlled conditions. Using this approach, we classified the virus responses of cassava lines from Africa and South America and identified truly virus-resistant clones that cannot be infected with any of the known viruses causing CBSD even under the most stringent virus infections. A modification of this protocol was used to test seedlings from cassava crosses for resistance against both diseases. A broad-spectrum resistance was identified in a workflow that lasted 9 months from seed germination to the identification of virus resistance. The workflow we propose dramatically reduces the evaluation and selection time required in a classical breeding workflow to reach the advanced field trial stage in only 9 months by conducting selections for virus resistance and plant multiplication in parallel. However, it does not bypass field evaluations; cassava resistance assessment prior to the field limits the evaluation to candidates with virus resistance defined as the absence of symptoms and the absence of the virus. The transfer of our virus screening workflow to cassava breeding programs enhances the efficiency by which resistance against viruses can be selected. It provides a precise definition of the plant’s resistance response and can be used as a model system to tackle resistance in cassava against other diseases.

## 1 Introduction

Viruses present a major threat to the cultivation of plants and, in particular, clonally propagated crops like potato, sweet potato, and cassava are menaced by a concoction of viruses from diverse genera. Host plant resistance is a key element of crop management but is limited by the availability of resistant sources. Breeding for virus resistance in a clonal crop is further complicated by the reproductive biology of the plant, the origin and inheritance of the genes conferring resistance, and the biology of the viruses threatening the crop.

In clonal plants, viruses are maintained and passed on to successive growing cycles through vegetative propagules. When plant propagation is not done *via* true seeds that clear viruses and effectively disrupt infection cycles, viruses become widely established within plant populations and evolve in uninterrupted plant infections. Thus, in vegetative crops, the challenge is to identify any resistance that interferes with the viral infection by blocking virus replication and efficiently preventing the establishment of an infection. As the virus does not replicate in a resistant plant, the infection is not carried over to the next growing cycle with vegetative propagules taken for planting. In disease-tolerant plants, viral infections are established but the diseases are associated with only a limited expression of symptoms and plant development is not critically impacted. Breeders and agronomists who assess losses from the disease set limits and thresholds for tolerance. However, since viral infections are maintained in successive cropping cycles, the tolerance assessment for a particular plant genotype may change over time because of the continuous use of virus-infected planting material that may lead to a higher incidence and severity of symptoms. Consequently, to be sustainable, disease-tolerant varieties require a strong seed system providing healthy planting materials.

Natural resistances against pathogens are mostly found in wild relatives of cultivated crops. Using such sources of resistance for breeding is often associated with major drawbacks concerning the genetic background of the progenitor carrying unwanted traits. Further impediments to rapid breeding progress are the inheritance of traits and the complex infection biology of the pathogen, complicating screening and selection of promising resistant candidates.

In this paper, we address the challenges associated with breeding cassava for resistance against the major viruses threatening the cultivation of the crop in Africa. We summarize the current knowledge of virus resistance in cassava and describe our approaches to accelerating resistance breeding by choosing defined sources of virus resistance and applying a precise virus infection workflow to shorten the virus screening processes in conventional breeding programs. The virus resistance we identified in cassava seedlings provides complete protection against the two most important cassava viral diseases in Africa, the cassava mosaic disease (CMD) and the cassava brown streak disease (CBSD).

## 2 Viruses infecting cassava in Africa

Two viral diseases, CMD and CBSD, caused by viruses from different families with distinct and diverse genomes and unique biological characteristics, threaten cassava in Africa. The major impact of these viral diseases is yield loss from severe symptoms on leaves leading to reductions in tuber sizes (CMD) (Thresh et al., 1994; Pita et al., 2001), necrosis of tuberous roots (CBSD) (Hillocks and Thresh, 2000; Kawuki et al., 2019), and plant decline (CMD & CBSD).

**Figure 1:**
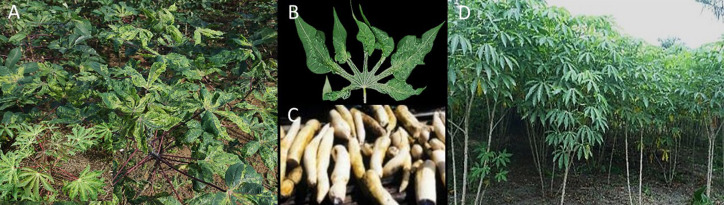
Severe mosaic symptoms of CMD **(A)**, leaf deformation **(B)**, and plant stunting in sensitive varieties result in small-sized root tubers **(C)**. The disease does not affect the highly resistant cassava variety TME 419 growing in an open field near Yangambi, DR Congo **(D)**.

The viruses causing CMD are endemic in Africa (Fondong, 2017) and the disease is present wherever cassava is grown on the continent. The cassava mosaic begomoviruses comprise distinct virus species (Patil and Fauquet, 2009; Legg et al., 2015) causing a similar disease in cassava (**Figures 1A–C**), and all are readily transmitted by the whitefly *Bemisia tabaci*. This makes controlling the diseases and restricting their spread in open fields challenging; thus, host plant resistance is the most effective disease control (**Figure 1D**).

**Figure 2:**
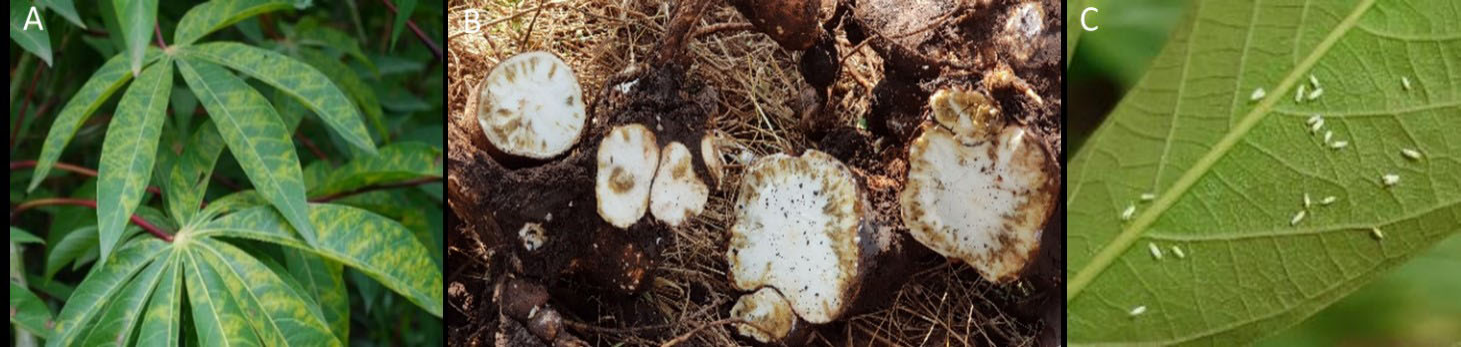
Vein chlorosis, and yellow blotches on leaves **(A)**, severe necrosis symptoms on tuberous roots **(B)** from CBSD on a sensitive cassava variety. Symptoms are mostly visible on older leaves and on tuberous roots reaching maturity. Semi-persistent virus transmission by the vector **(B)** *tabaci* **(C)** depends on high numbers of adult insects.

The viruses causing CBSD are the constituents of the current pandemic across East African countries, with epicenters in Uganda, Tanzania, Kenya, and Mozambique, and extending to neighboring countries (DR Congo and Zambia), where they present acute threats to cassava cultivation. Plant growth and development generally remain unaffected by the disease identifiable by characteristic leaf symptoms on older leaves (**Figure 2B**). However, the viruses cause root necrosis, and this destruction of the tubers renders them inedible (**Figure 2A**) (Nichols, 1950; Hillocks et al., 2001). The two distinct ipomovirus species causing CBSD, the cassava brown streak virus (CBSV) and the Ugandan cassava brown streak virus (UCBSV) (Winter et al., 2010), have complex infection strategies (Sheat et al., 2021) which complicate virus diagnosis and assessment of the disease. The viruses are inefficiently transmitted by *B. tabaci* (Maruthi et al., 2005) (**Figure 2C**) and their spread is bound to seasons with high whitefly populations. Human-assisted spread is the main pathway for their distribution. Although phytosanitary options exist, genetic resistance against the viruses causing CBSD is key to the cultivation of healthy cassava and preventing further spread and transboundary movement of the disease.

### 2.1 Recovery resistance and immunity in cassava against begomoviruses causing cassava mosaic disease

Breeding for virus resistance in cassava goes back to the Amani breeding program in Tanzania. It started in 1937 (Jennings, 1957), when CMD resistance from the wild relative *Manihot glaziovii* was introgressed in African cultivars. Seeds (clone 58308) from this interspecific hybrid backcrossed against *M. esculenta* were used extensively in the IITA breeding program, resulting in TMS 3001, TMS 30395, and TMS 30572 (Hahn et al., 1980). The improved cassava varieties had resistance against CMD, showed good breeding values, and, consequently, were widely distributed throughout the cassava regions of Africa. Their inherent CMD1 resistance, originating from *M. glaziovii*, is polygenic and recessive (Akano et al., 2002). CMD1 cassava lines can become infected with the virus but respond with milder symptoms and some eventually recover from symptoms and appear healthy while the infection persists. However, CMD1 resistance does not sufficiently protect against the species and strains of East African cassava mosaic virus (EACMV) now prevalent in East and Central Africa. CMD1 varieties respond with more severe symptoms to EACMV species and strains and do not recover from the disease.

Intensive search efforts led to the discovery of virus resistance in the west African landrace TME 3 (Akano et al., 2002), which provides a high level of resistance against many African and East African cassava mosaic viruses that can completely protect cassava against begomovirus infections. This CMD2 resistance has a dominant inheritance and is found in TME 204 (TME 419, Obama), Albert, Nsansi (TME 96/0160), Tz130 (NaroCASS1), and many other cassava lines and varieties. Today this is the basis of begomoviruses resistance in cassava.

In our laboratory, we infected several cassava lines carrying CMD2 with a broad range of begomovirus isolates, including African cassava mosaic virus, East African cassava mosaic Cameroon virus, East African Cassava virus-Uganda, and Sri Lankan cassava mosaic virus, and found that begomoviruses could not establish themselves in CMD2 cassava lines. This immunity was effective against all known begomoviruses and, although leaf symptoms on a few leaves initially developed on some cassava lines, virus replication was not further supported and the plants remained symptom-free and free of the virus. Despite its monogenetic nature and its wide use in modern varieties, the resistance provided is robust; during more than 20 years of its use, resistance breaking has never been observed. Recent evidence suggests that the outstanding characteristics of CMD2 resistance in cassava are associated with mutations in the DNA polymerase δ subunit 1 (MePOLD1) located within the CMD2 locus on chromosome 12 (Lim et al., 2022).

Introducing CMD2 to confer begomovirus resistance in cassava is an ideal breeding target because the resistance is clearly defined. High-performing African varieties with CMD2 resistance are available and breeding tools are on hand to support a controllable and reproducible screening process (Okogbenin et al., 2012; Rabbi et al., 2014; Thuy et al., 2021).

### 2.2 Tolerance in cassava against ipomoviruses causing cassava brown streak disease

Cassava brown streak disease has been known for a long time, and an early report on CBSD in Tanzania (Storey, 1936) was swiftly followed by a resistance breeding program at the Amani research station (Jennings, 1957) despite the causal agent(s) of the disease not being known (Hillocks and Thresh, 2000). Similar to CMD, inter-specific hybrids with wild relatives of *M. esculenta* were generated and one offspring of the program, clone 46106/27, also known as Namikonga (syn. Kaleso), became an important source of CBSD resistance. Namikonga was less affected by the disease and developed only moderate leaf symptoms and limited necrosis on tuberous roots. Namikonga was considered tolerant, and when CBSD-resistance breeding intensified in the early 2000s, Namikonga was incorporated into many crosses like NASE 1 and NASE 14 (Kawuki et al., 2016). In recent years, cassava lines with resistance against U/CBSV have been developed that show only mild symptoms on leaves and stems when infected and much less root necrosis (Jennings, 1957; Kaweesi et al., 2014; Kawuki et al., 2016; Masinde et al., 2018; Mukiibi et al., 2019). Nevertheless, despite progress to enhance the level of tolerance, cassava genotypes with high levels of resistance have not yet been found (Bart and Taylor, 2017).

We infected cassava germplasm from South America and cassava varieties from Africa with well-characterized virus isolates (Sheat et al., 2019) and followed the virus infections over many months. We confirmed earlier reports (Ogwok et al., 2014) on the high sensitivity of NASE 14 and NASE 3 to CBSV and showed that KBH 2006/18 and KBH 2006/26 (Mkuranga), two varieties that were considered immune (Anjanappa et al., 2016), can in fact be infected with such viruses (Sheat et al., 2019). Finally, we concluded that all African cassava varieties were susceptible to the viruses and responded to the disease with mild to severe leaf symptoms (**Table 1**).

**Table 1:** Response of cassava lines and varieties upon infection with CBSV and UCBS.

Name/accession	CBSV	UCBSV
KBH 2016B/504	S0	S+
KBH 2016B/185	S++	S+
KBH 2016B/521	S+	S++
KBH 2016B/020	S+++	S+
KBH 2016B/087	S+++	S+
Yizaso	S+++	not tested
Eyope	S+++	not tested
NaroCASS1 (TZ 130)	S++	S+
Orera	S++	not tested
Mkuranga (KBH 2006/26)	S+++	S+
Kipusa (KBH 2002/066)	S+++	S+
Mkumbozi (MM96/4684)	S+	S+
Pwani (B2c20-65)	S++	S+
Mkumba (3C20-10)	S+	S+
Kizimbani	S+++	S+
Kiroba	S+	S+
Nase 19 (72-TME14)	S+++	S+
Nase 1 (TMS 60142)	S+	not tested
Nase 3 (TMS 30572)	S++	S0
Nase 14 (MM192/0248, MM96/4271)	S+++	not tested
TME 419 (TME204, Obama)	S++	S++
MM2006/0123	S+	S+
MM2006/0128	S+	S+
NaroCASS2 (MM2006/0130)	S+	S+
UG120198	S+	S+
UG120001	S+	not tested
UG120024	S+	S+
UG120156	S+	S+
Game changer	S+	not tested
Poundable (TME 693)	S+	not tested

S, sensitivity status; +++, very severe leaf symptoms, deformation, wilting, plant death; ++, severe leaf symptoms; + mild to moderate leaf symptoms; 0, plant cannot be infected.

In our infection studies, we recorded pronounced differences in plant responses against the two viruses, the most striking being disease progress. While CBSV symptoms developed within weeks after grafting, it could take many months (6-8 months), even under stringent virus infection conditions, before UCBSV symptoms became evident. Moreover, it could even take much longer before root necrosis symptoms became visible. Secondary plant infections, from infected cuttings, showed root necrosis earlier because the higher virus loads in persisting virus infections led to early tissue necrosis in developing tubers that increased along with secondary growth. Thus, the extent of root necrosis was correlated with the length of infection and the species of the virus.

Furthermore, virus species-specific responses were recorded for several cassava genotypes. The popular variety, “Mkuranga” responded with mild symptoms when infected with UCBSV but showed severe leaf symptoms and wilting when infected with CBSV. TMS 30572 was highly sensitive to CBSV but this genotype could not be infected with UCBSV. In contrast, the breeding line KBH 2016B/504 could not be infected with CBSV (**Table 1**) but was sensitive to UCBSV. Both cassava genotypes could be highly interesting sources of resistance; however, their potential was not evident when the diseases were evaluated without resolving the causal viruses. Thus, knowledge about the virus species present in a particular genotype is a prerequisite for reproducible and comparable resistance/tolerance evaluations.

Kaweesi et al. (2014) evaluated the response of NASE 14 to CBSD infection in the field and recorded in some plants (15) a complete absence of symptoms, while others (4) showed mild leaf symptoms only, and two plants had a high incidence and severity of necrosis symptoms on tuberous roots. The latter observation indicated that this variety was highly susceptible to CBSD but may not become readily infected; thus, the absence of symptoms may be due to a lack of infection. In any case, such variations cause uncertainties concerning the assessment of CBSD disease/tolerance due to the lack of control over virus infection processes. In field situations, the transmission of U/CBSV was highly erratic and the time points of the virus infections could vary dramatically between each individual plant, which then led to highly variable root necrosis symptoms and severity scores.

The virus infection and screening protocol we adopted in our laboratory reduced the uncertainties associated with virus species and time point of infection. A highly effective plant infection with a known virus isolate ensured that almost 100% of the plants become infected at a given time point. This assured that plant responses against the viruses are reproducible.

**Figure 3:**
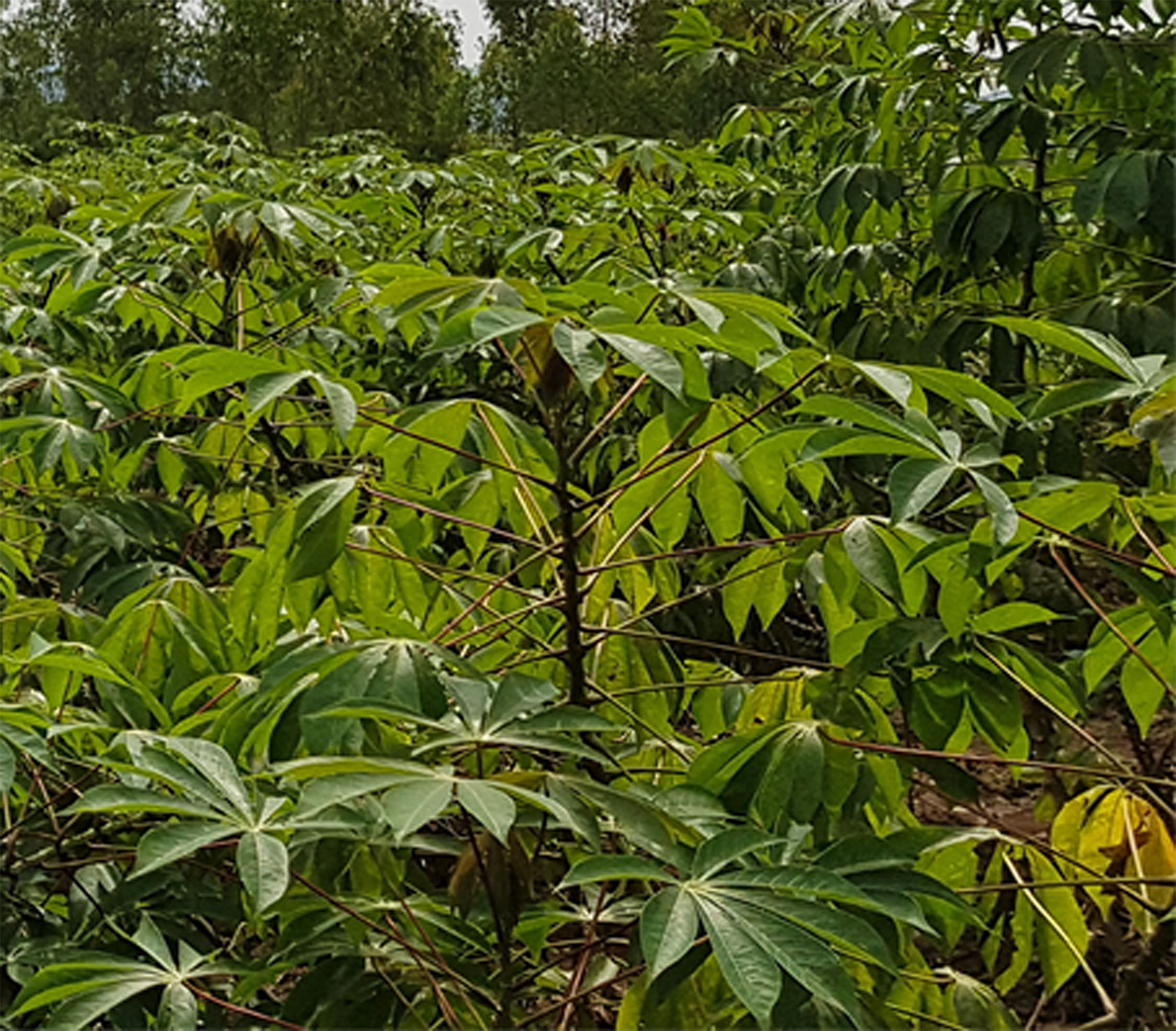
The advanced breeding line KBH 2016B/521, 6 months after planting in an epidemic zone for CBSD and CMD near Uvira, DR Congo. The line is free of symptoms after 3 consecutive growing seasons.

In all our experimental infections, and complemented by several years of field trials in DR Congo, the advanced breeding lines KBH 2016B/504 and KBH 2016B/521 have shown extraordinary resilience against U/CBSV (**Figure 3**). Although susceptible to the viruses, it was very difficult, even under our stringent conditions, to establish infections. Only limited CBSD symptoms were found on leaves, and root necrosis eventually developed but was limited to small areas of the roots only. The plants are highly resistant to CMD and show good field performance, which emphasizes their potential as parents for further cassava improvement.

### 2.3 Immunity, differential resistance, and tissue-specific resistance in cassava against ipomoviruses causing cassava brown streak disease

Our search for new sources of U/CBSV resistance in South American cassava germplasm (Sheat et al., 2019) was motivated to find cassava lines that could not be infected by any U/CBSV isolate, thus expressing an immunity status similar to the begomovirus response provided by CMD2. This would create complete protection against CBSD and resolve ambiguities associated with categories of tolerance, breaking of tolerance, and concerns about persisting virus infections in clonal crops.

In a stringent virus screening workflow applied to approximately 300 cassava germplasm lines of the CIAT collection, we infected cassava plants with the most severe CBSV isolates (DSMZ PV949; FN434436), and those with virus symptoms and testing positive by qRT-PCR were eliminated. Plants that stayed healthy were further subjected to infections with UCBSV.

Only three lines passed this stringent virus screening, and high resistance against U/CBSV viruses was identified in COL 40, COL 2182, and PER 556 (**Table 2**), the first two varieties originating from Columbia and the latter from Peru. Even under high virus pressure from a grafted virus-infected branch, these cassava lines did not become infected. There were no symptoms expressed and no virus was detected in any tissue. U/CBSV remained in the phloem companion cells and there was no virus replication (Sheat et al., 2019; Sheat et al., 2021). We identified further cassava lines (e.g. PER 333 and PER 353) that restricted U/CBSV to the roots associated with necrosis symptoms, while leaves remained free of symptoms and virus infection. In this case, the virus replicated in phloem companion cells but was not able to translocate to adjacent parenchymatic tissues of stems for replication (Sheat et al., 2019; Sheat et al., 2021).

**Table 2:** Cassava germplasm from South America with resistance against cassava brown streak viruses.

DSMZ acronym.	CIAT accession	CBSV status	UCBSV status
DSC 118	COL 40	resistant	resistant
DSC 167	COL 2182	resistant	resistant/susceptible^*^
DSC 196	ECU 41	resistant	susceptible
DSC 250	PER 221	resistant	susceptible
DSC 269	PER 556	resistant	resistant
DSC 120	COL 144	resistant	susceptible
DSC 258	PER 333	resistant	root restricted
DSC 199	ECU 159	root restricted	susceptible
DSC 257	PER 315	root restricted	susceptible
DSC 260	PER 353	root restricted	root restricted
DSC 261	PER 368	root restricted	not tested
DSC 272	PER 597	root restricted	susceptible
DSC 122	COL 262	root restricted	susceptible
DSC 248	PER 206	root restricted	susceptible
DSC 251	PER 226	root restricted	susceptible
DSC 186	CUB 40	susceptible	susceptible
DSC 142	COL 1107	susceptible	not tested

^*^Under specific experimental conditions, the line can be infected with UCBSV only (Sheat et al., 2021).

The cassava line COL 40 has been subjected to field infections for several growing cycles and, so far, U/CBSV have never been detected, emphasizing the outstanding resistance performance of this line. Similarly, the resistance against U/CBSV identified in the South American cassava germplasm accessions PER 556 and COL 2182 (**Table 3**) is considered plant immunity: the lines do not support virus replication, and the cassava brown streak disease does not establish.

**Table 3:** Cassava crossing populations generated at CIAT and IITA with U/CBSV-resistant parents.

Population Nr.	Mother	Father
1 CIAT	PER 353	GM 7673-3
	GM10054B-1	PER 221
	GM10054B-1	PER 353
	GM10054B-2	PER 353
	GM10055B-2	PER 353
	GM10062-1	PER 353
	C 33	PER 221
	C 33	PER 353
	C 39	PER 353
	C 243	PER 353
	C 413	PER 353
	COL 40	**C 33**
	COL 144	GM 7673-3
	COL 144	GM10055B-1
	COL 144	GM10055B-2
	COL 144	C 19
	COL 144	**C 33**
	COL 144	C 39
2 IITA	KBH 2016B/504	TME 14
	COL 40	KBH 2016B/185
	COL 40	KBH 2016B/087
	COL 40	TME 14
3 CIAT	COL 144	C 19
	COL 144	C 39
	GM6127-15	PER 221
	GM7672-8	PER 221
	COL 40	GM6127-13
	COL 144	TME 3
	COL 40	NN
	ECU 41	NN
4 IITA	COL 40	KBH 2016B/504
	COL 40	TME 14
	COL 40	MM2016/1487

COL 40 crosses provide broad-spectrum resistance against all U/CBSV viruses. C33, TME3, and TME14 have proven resistances against cassava mosaic viruses.

## 3 Breeding for resistance against cassava mosaic viruses and cassava brown streak viruses

**Figure 4:**
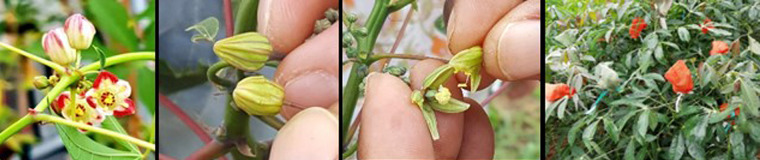
Cassava flowers: unusual colors mark the flowers of some South American x African cassava crosses (left), COL 2182 parents setting seeds at the TARI crossing block in Maruku, Tanzania.

The South American cassava lines selected for U/CBSV resistance (**Table 3**) were very sensitive to CMD and instantly became infected with severe disease symptoms. Thus, our NextGen Cassava partners, CIAT (Columbia), IITA (Uganda), and TARI (Tanzania) used the new sources of CBSD resistance to generate crosses between South American and African lines (**Figure 4**) to also include the most promising CBSD resistant lines COL 2182, PER 556, and COL 40. The latter is currently the most widely used CBSD parent because of its readiness to flower and its resilience to CMD in Africa.

### 3.1 Immunity against U/CBSV is expressed in F1seedling populations

Seedlings from crosses comprising U/CBSV resistant parents (**Table 3**) were subjected to a stringent virus-resistance screening (Sheat et al., 2022). Since the resistance status of both seedling parents was known, we modified our resistance discovery workflow (Sheat et al., 2019) and adopted a more rapid virus screening process that would identify/confirm virus resistance/susceptibility in seedlings within a few weeks if this resistance phenotype was evident in F1 seedlings.

The high throughput virus screening workflow consisted of two cycles (identification and confirmation). In the first cycle, we grafted scions from CBSV-infected plants to infect each seedling. Seedlings that showed virus symptoms and tested positive with qRT-PCR (Sheat et al., 2019) were eliminated from further testing. Seedlings that tested negative were grafted with scions of cassava plants that were mixed-infected with UCBSV and EACMV-UG (GenBank accessions UCBSV, MW961202; EACMV-UG OL44492, OL444943), as described in (Sheat et al., 2022). All plants that passed cycle two and tested negative by qRT-PCR and qPCR for U/CBSV and EACMV entered the confirmation cycle, in which all steps of the workflow were repeated with a higher number of plants. Along with the confirmation cycle, the resistant cassava candidates were transferred and established in African fields to evaluate virus resistance under natural conditions. All experiments, including molecular testing, are described in detail (Sheat et al., 2019). A graphical overview of this workflow and further descriptions can be found in (Sheat et al., 2022).

The seeds obtained from the breeding programs (**Table 3**) included crosses with: COL 40, providing complete immunity against U/CBSV; PER 221, which has a differential resistance against CBSV; and PER 353, in which U/CBSV remains restricted to the roots. Complete control over CBSD can only be reached when COL 40 is used as a CBSD parent. However, crosses with other parents can provide insights into the resistance mechanisms.

Infecting seedlings from population 1 crosses (**Table 3**) with CBSV resulted in virus infections that became readily evident with symptoms developing within six weeks after grafting. Several seedlings in population 1 families did not become infected with CBSV; there were no symptoms indicating for virus infection and the virus was not detected. As the resistance phenotype was visible in the F1 population, we can assume that the CBSD resistance we identified in South American germplasm is a dominant trait.

Virus infection assays comprising further populations (**Table 3**) and higher numbers of seedlings are still ongoing to assess inheritance from infecting a large number of seedlings from different crossing families (**Table 3**). However, it is already clear that a resistance phenotype expressed in F1 as a binary response radically facilitates selection processes and speeds up resistance breeding.

### 3.2 Broad spectrum immunity against viruses causing CMD and CBSD

We screened for resistance against both diseases by subjecting the CBSV-resistant seedlings to further infections with UCBSV and a severe isolate of East African cassava mosaic virus (EACMV-UG). As COL 40 is immune to all U/CBSV isolates, UCBSV testing was taken as further confirmation of the broad spectrum of the resistance.

Graft transmission of the cassava mosaic virus resulted in infections in susceptible seedlings within 3 to 4 weeks. In unclear symptomatology, excision of the apical parts of a graft-infected plant (comprising the first three leaves) provoked new leaf flushes, along with the expression of pronounced symptoms. A persistent symptomatic phase verified a cassava mosaic virus infection. Longer observation times were needed to identify true CMD resistance in seedlings because resistant plants can initially develop symptoms on a few leaves but thereafter recover, with new leaves free of both symptoms and the virus.

In this first screening for dual resistance against viruses causing CBSD and CMD, we identified five seedlings from the 18 families of population 1 (**Table 4**) having complete immunity. The plants stayed healthy and virus-free even under high virus pressures from grafted virus-infected scions.

**Table 4:** Cassava seedlings (population 1) with resistance against viruses causing CMD x CBSD.

Mother	Father	Nr. of resistant seedlings	Name
PER 353	GM 7673-3	1	1-1 (DSC 673)
C 33	PER 221	2	8-1, 8-10 (DSC 516, DSC 525)
COL 40	C 33	1	12-1 (DSC 493)
COL 144	C 39	1	18-8 (DSC 510)

From our predictions, only seedling 12-1 (**Table 4**) carries the broad-spectrum U/CBSV resistance from its COL 40 parent. Even after 18 months of infection, the four seedlings with predicted sensitivities to UCBSV did not show UCBSV symptoms and the virus was never detected.

While this warrants further explanation, it also discloses a weakness of the glasshouse-based virus testing. While this virus screening is very powerful for rapidly identifying virus-susceptible plants, proof of virus resistance/immunity can only be comprehensively provided when tuberous roots are tested. This is very difficult to achieve under screen/glasshouse conditions; hence, a confined field trial with infected plants at early screening stages is needed to provide further clarifications on the fate of the virus in an infected plant and on the immunity status of the genotype.

Phenotyping for virus resistance becomes more complex and lengthier when the chosen parents have a level of tolerance against U/CBSV. This is because the infection processes are dramatically delayed and thus viral infection and phenotyping of the best-predicted crossing combinations, e.g., COL 40 x KBH 2016B/504 (**Table 3**, 4 IITA), may require controlled infections in the field followed by prolonged observation times. However, because seedlings from virus-sensitive crosses have already been eliminated, the efforts can focus on fewer final candidates.

**Figure 5:**
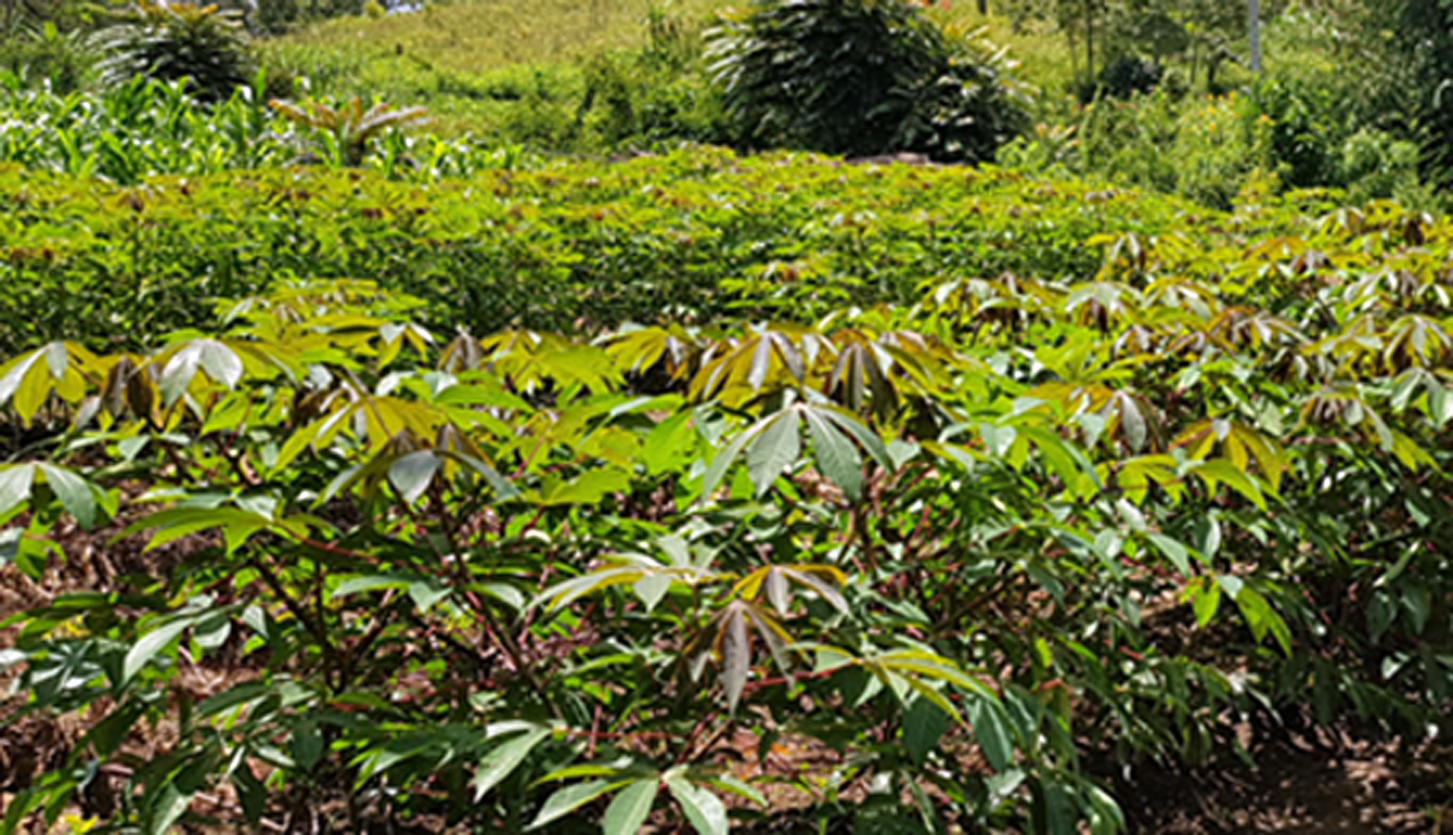
Cassava seedling DSC 493, 6 months after planting in an epidemic zone for CBSD and CMD near Uvira, DR Congo. The line is free of symptoms and shows vigorous growth.

The 12-1 seedling (DSC 493) (**Figure 5**) and the other four candidates selected from population 1 (**Table 4**) are currently being subjected to confirmation-round testing under greenhouse conditions. At the same time, the lines are being grown at three cassava stations in Africa (DR Congo AVPD, DR Congo IITA, and Tanzania IITA) to check for their resistance status and agronomic performance under natural conditions to ultimately prove their potential to mitigate the impact of CMD and CBSD in Africa.

## 4 Screening for resistance against viruses causing CBSD X CMD in the field

Several strategies can be followed to accelerate virus-resistance screening under field conditions. The high throughput screening protocol (Sheat et al., 2022) we developed solved the main uncertainties associated with U/CBSV resistance assessment in cassava from uncontrolled and erratic infection processes in the field. When CBSD x CMD crosses were tested (3.2), the screening started with CBSV infections of seedlings because we assumed that the parents, including COL 40, PER 221, and PER 353, would either be highly resistant or highly sensitive to CBSV. When such crosses are tested under field conditions in Africa, it can be assumed that the seedlings are either highly resistant to CMD begomoviruses from C33 and TME14 parents, or highly sensitive because South American cassava varieties lack this resistance. As whitefly transmission of CMD begomoviruses is very efficient in the field and sensitive seedlings from CBSD x CMD crosses react rapidly and with pronounced symptoms, the first step of field screening comprises monitoring of CMD symptoms to eliminate susceptible seedlings. All seedlings that did not become naturally infected are then infected by grafting with U/CBSV. The U/CBSV used for infections are sequence-characterized viruses representing the isolates predominant in the region. As such, a set of resistant candidates is created that can be subjected to further infection experiments with other virus combinations for confirmation. Resistance testing of CBSD x CMD crosses under field conditions is feasible when appropriate conditions for U/CBSV infections are established. It requires a seedling nursery, a propagation plot to maintain virus-infected cassava source plants, and a screenhouse for virus infections and to protect sensitive seedlings and rootstocks during the first weeks after grafting. When complemented with a limited laboratory infrastructure for virus testing, cassava virus resistance screening in the field converts to a precise and reproducible process to accelerate breeding.

## 5 Future perspectives

Resistance against the two most widely distributed and severe virus diseases, cassava mosaic disease and cassava brown streak disease, is a prerequisite for growing a healthy and productive crop in Africa. Considering the geographic extension of CBSD on the continent and the current invasion of the viruses causing CMD to spread into new regions in South East Asia, Cambodia, Vietnam, and Thailand, the incorporation of resistance to viruses should be a global requirement for cassava just like it is for the potato (*Solanum tuberosum* L.). Therefore, developing cassava with resistance against these two viral diseases is a response to the acute threat of CBSD and a preemptive measure for regions at risk. The CMD2 resistance from African cassava that protects from CMD also presents the cure against the Sri Lankan Cassava mosaic virus causing CMD in South East Asia.

CMD2 provides complete resistance against all currently known begomoviruses infecting cassava. This high resistance is considered immunity because an infecting virus cannot establish itself and the infection is aborted. Furthermore, there are no reports of resistance-breaking viruses; hence, this resistance appears to be broad-spectrum and durable. A likely explanation is that a vital interaction and critical element for geminivirus replication is disrupted.

The resistance to viruses causing CBSD in South American germplasm lines blocked cassava brown streak viruses from replication and confined the pathogens to the phloem companion cells. There is no evidence so far that the viruses can establish themselves in a plant when grafted with scions of infected plants. The resistant plants were grown in the field under disease pressure without developing symptoms or viral infections. However, because CBSD resistance was only recently found (Sheat et al., 2019) and characterized (Sheat et al., 2021), nothing is known about its mechanism and, more critically, no field data have been collected over several seasons, including assessments of tuberous roots. COL 40 provides strong resistance against a broad range of U/CBSV isolates; however, we need to consider the limited repertoire of isolates tested and the rather short time of observation. Long-term data do not exist and we cannot rule out that viruses, variants, and/or strains may not appear that escape the resistance response, accumulate in vegetative cycles, and cause disease. This can only be elucidated in virus studies accompanying field trials of CBSD x CMD crosses.

Cassava brown streak disease is a very complicated disease because of its infection biology and its impact on tuberous roots. The rapid disease phenotyping approach we have developed solves major impediments in resistance screening by providing a defined workflow for virus infection and testing. By subjecting seedlings from resistance crosses to this workflow, precise phenotyping and identification of CBSV-resistant genotypes have been made possible that provide the fundament for a genome-wide association study (GWAS) to further our understanding of the resistance and to guide future breeding.

The first generation of prototypes with CBSD x CMD immunity was selected from crosses with South American and African germplasm using our high-precision virus screening (Sheat et al., 2022). Indeed, the limited number of resistant plants does not represent a diversity sufficient for breeding populations, but does provide the resistances for further breeding. As this method is highly efficient and precise in identifying resistant candidates, it will even be more useful when advanced crosses between highly CBSV-tolerant parents (e.g., KBH 2016/504) and CBSV-immune lines (e.g., COL 40) and their progenies are to be tested.

There is no doubt that the effective and precise workflow developed for CBSD-resistance evaluation will accelerate resistance breeding. Its future lies in the transfer of the concept and methods to breeding sites. This requires a change of perspective regarding how screening and selection for virus resistance in cassava is done, but success is one step closer when the field is considered an open laboratory space.

## Data availability statement

The raw data supporting the conclusions of this article will be made available by the authors, without undue reservation.

## Author contributions

Conceptualization, methodology, validation, writing, review, and editing: SS, SW. All authors contributed to the article and approved the submitted version
